# Investigation of Thiol/Disulfide Homeostasis and Clinical Parameters in Rosacea Patients According to Skin Subtypes

**DOI:** 10.3390/jcm13144052

**Published:** 2024-07-11

**Authors:** Nilufer Yesilirmak, Ozge Saritas, Busra Kurt, Salim Neselioglu, Akin Aktas, Ozcan Erel

**Affiliations:** 1 Department of Ophthalmology, Ankara Yildirim Beyazit University, 06800 Ankara, Turkey; 2 Department of Medical Biochemistry, Gazi University, 06500 Ankara, Turkey; 3 Department of Ophthalmology, Ankara Bilkent City Hospital, 06800 Ankara, Turkey; bukocabas@gmail.com; 4 Department of Ophthalmology, Battalgazi State Hospital, 44320 Malatya, Turkey; ozzgesaritass@gmail.com; 5 Department of Medical Biochemistry, Ankara Yildirim Beyazit University, 06800 Ankara, Turkey; sneselioglu@ybu.edu.tr (S.N.); erelozcan@gmail.com (O.E.); 6 Department of Dermatology, Ankara Yildirim Beyazit University, 06800 Ankara, Turkey; akaktas@hotmail.com

**Keywords:** rosacea, rosacea skin subtypes, ocular rosacea, oxidative stress, thiol/disulfide homeostasis, clinical parameters

## Abstract

**Background/Objective:** The aim of this study was to compare thiol/disulfide homeostasis and clinical parameters of rosacea patients across skin subtypes of the disease and healthy controls. **Methods:** This prospective study included 90 rosacea patients with different skin subtypes (phymatous, erythematotelangiectatic and papulopustular) and ocular involvement and 30 healthy controls. Plasma native thiol (NT), total thiol (TT) and disulfide levels of the patients and controls were measured using an automated spectrophotometric method, and disulfide/native thiol ratio (DNTR), disulfide/total thiol ratio (DTTR) and native thiol/total thiol ratio (NTTR) were calculated. Tear breakup time (TBUT), meiboscore, Schirmer, ocular surface disease index (OSDI) and rosacea-specific quality of life scale (RosaQoL) were measured clinically. **Results:** Disulfide, DNTR and DTTR were significantly higher, and NT, TT and NTTR were significantly lower in the rosacea patients compared to the controls (*p* < 0.001). TBUT and Schirmer were significantly lower, and meiboscore and OSDI were significantly higher in the patients compared to the controls (*p* < 0.01). According to the skin subtypes, disulfide, DNTR and DTTR were significantly higher, and NTTR was significantly lower in the erythematotelangiectatic subtype compared to the other subtypes (*p* < 0.002). TBUT was significantly lower, and RosaQol was significantly higher in the erythematotelangiectatic subtype (*p* < 0.0083). Strong correlations were found between DNTR and TBUT and between DNTR and Meiboscore in all subtypes (*p* < 0.005), while there were strong correlations between DNTR and OSDI and between DNTR and RosaQol only in the erythematotelangiectatic and papulopustular subtypes (*p* < 0.05). **Conclusions:** The thiol/disulfide homeostasis shifted towards disulfides, an indicator of oxidative stress in rosacea, and this was more pronounced in the erythematotelangiectatic subtype. The impairment in TBUT and RosaQol was also more prominent in the erythematotelangiectatic subtype and strongly associated with the DNTR.

## 1. Introduction

Rosacea is a chronic, common, inflammatory disease with telangiectasia, pustules, and papule changes, particularly on central facial skin [1,2]. By the National Rosacea Society Expert Committee, skin rosacea is divided into three subtypes based on the dominant phenotype: phymatous, erythematotelangiectatic and papulopustular [3]. Ocular rosacea occurs in more than half of the patients before or after skin involvement [4,5].

Ocular involvement of rosacea generally presents with blepharitis, meibomian gland dysfunction, lid margin telangiectasia and interpalpebral conjunctival injection [6,7]. In advanced cases, it may cause serious visual impairment with corneal and scleral involvement [6]. As in skin rosacea, the pathogenesis of ocular rosacea is also complex and still not fully known. In recent years, it has been shown that dysregulation of inflammatory, vascular, immune and neuronal systems; genetic predisposition; demodex colonization; ultraviolet radiation (UV) and oxidative stress play a role in its pathogenesis [8,9,10].

In skin rosacea, it has been indicated that demodex antigens stimulate the generation of reactive oxygen species (ROS) by releasing proinflammatory cytokines and increasing the number of neutrophils [11]. Other studies also confirmed that oxidative stress plays a role in the emergence of rosacea lesions in skin through a number of pathways, such as neutrophil-produced ROS, protein and lipid peroxidation and immune system stimulation [12,13]. It has been shown that ROS trigger vascular alterations and inflammation in skin rosacea and are associated with the oxidative modification of lipids and proteins [14,15]. However, the role of oxidative stress in ocular rosacea was not clear. In a very recent study, it has been concluded that oxidative stress seems to play significant and concurrent roles in both ocular and skin rosacea [16].

Several markers can be used to assess the level of oxidative stress in biological systems, such as thiol-disulfide homeostasis [17,18]. Thiols (-SH) are organic compounds made up of hydrogen and sulfur atoms. Thiols act as very potent antioxidants since they reduce unstable free radicals. Disulfides (-S-S-) are dynamic covalent bonds formed between two thiol groups [18]. In oxidative stress conditions, hydrogen is released from thiols and forms disulfide bonds. And released hydrogen binds excess oxygen for the deactivation of ROS. Diseases, especially those marked by chronic inflammation, appear to have altered thiol-disulfide balances towards disulfides [19,20]. Similarly, in skin rosacea patients, the thiol/disulfide balance has also been shown to shift towards disulfides, as an indicator of oxidative stress [21,22,23]. However, to the best of our knowledge, none of the previous studies have investigated thiol/disulfide homeostasis in rosacea patients with ocular involvement as well as how these levels are associated with clinical parameters and change according to rosacea skin subtypes.

In our study, we aimed to evaluate thiol/disulfide homeostasis and rosacea-related clinical parameters of both ocular- and skin-involved rosacea patients across skin subtypes of the disease and to compare those with each other and with healthy controls. In addition, we aimed to explore the association of thiol/disulfide homeostasis with ocular surface parameters.

## 2. Materials and Methods

### 2.1. Study Participants

This prospective, comparative study involved rosacea patients (n: 90) diagnosed with different skin subtypes (as phymatous, erythematotelangiectatic and papulopustular) by the examination of a senior dermatologist (A.A.) and confirmed having mild–moderate ocular rosacea (lid margin telangiectasia, blepharitis, blepharoconjunctivitis and meibomian gland dysfunction) by the examination of a senior ophthalmologist (N.Y.). Thirty age- and gender-matched healthy voluntaries were included as controls. The Ethics Committee of Ankara Bilkent City Hospital approved this study, and informed consent was received from the participants; this study adhered to the tenets of the Declaration of Helsinki.

### 2.2. Exclusion Criteria

Patients having a history of other systemic or ocular disease, ocular rosacea with a severe course (corneal involvement), systemic or ocular medication, supplements, pregnancy, alcohol use and cigarette use were excluded from this study.

### 2.3. Clinical Data Collection

**Figure 1:**
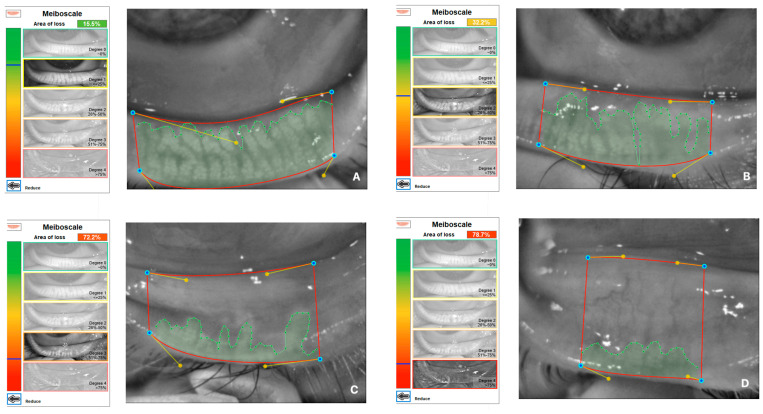
Exemplary meibography images demonstrating meiboscore of patients. (**A**) A patient with Grade 1 meiboscore (gland loss rate is 15.5%). (**B**) A patient with Grade 2 meiboscore (gland loss rate is 32.2%). (**C**) A patient with Grade 3 meiboscore (gland loss rate is 72.2%). (**D**) A patient with Grade 4 meiboscore (gland loss rate is 78.7%).

**Figure 2:**
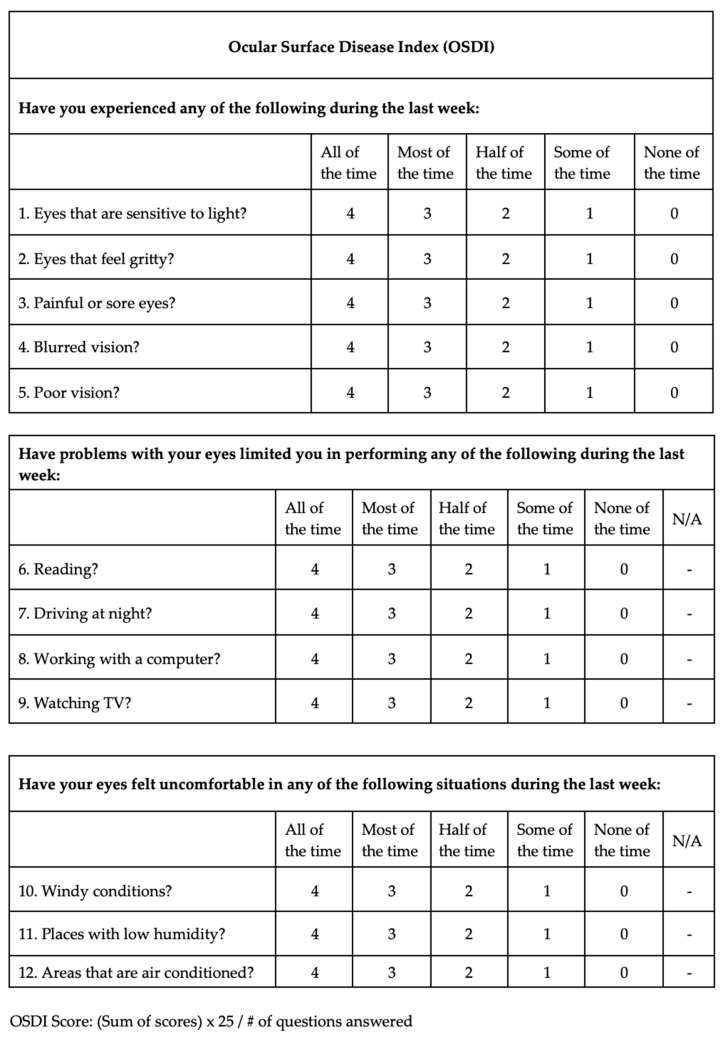
Ocular surface disease index (OSDI) questionnaire and scores.

**Figure 3:**
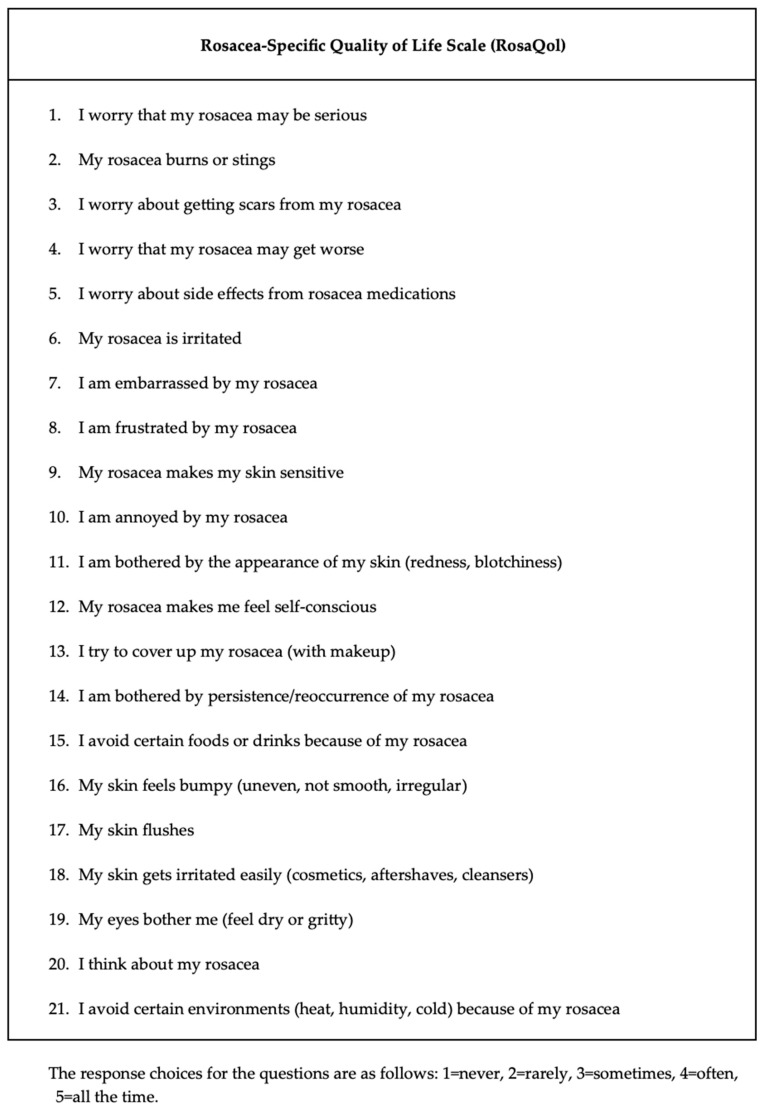
Rosacea-specific quality of life scale (RosaQol) questionnaire and scores.

Demographics, ophthalmological examination features and rosacea-related clinical parameters (tear breakup time (TBUT), Schirmer-1 test results, ocular surface disease index (OSDI) score, meibomian gland dysfunction score (meiboscore) and rosacea-specific quality of life scale (RosaQol) score) were recorded from all participants. TBUT was measured after placing 5 μL of fluorescein into the conjunctiva. After blinking several times, the time between the last blink and the presence of the first black dot on the cornea was noted [24]. To perform the Schirmer test, a Schirmer strip was placed on the lateral canthus of the lower lid, and the amount of wetting on the strips in 5 min was recorded in millimeters [25]. The meiboscore was measured [26] by obtaining infrared meibography images of the lower eyelids with a Sirius topography device (Sirius, CSO, Florence, Italy) and grading the rate of loss in the glands from 0 to 4 (<10% loss = Grade 0; 10–25% loss = Grade 1; 25–50% loss = Grade 2; 50–75% loss = Grade 3; >75% loss = Grade 4). Exemplary meibography images demonstrating the meiboscore of the patients are given in Figure 1. OSDI scores were obtained by scoring, on a scale from 0 to 100, 12 items in the OSDI questionnaire [27], which are based on the patients’ symptoms related to the condition of the ocular surface (Figure 2). RosaQol scores were obtained by scoring, on a scale from 21 to 105, 21 items in the questionnaire [28] assessing rosacea-specific quality of life that assesses symptoms, functioning and emotions (Figure 3).

### 2.4. Serum Collection

Blood samples were collected in biochemical tubes after 8 h of fasting. The blood samples were promptly centrifuged at 4000 rpm for 10 min, and then, serum parts were placed in 1.5 mL Eppendorf tubes and stored at −80 °C until analysis.

### 2.5. Biochemical Analysis

All serum samples were analyzed simultaneously. Plasma native thiol (NT), total thiol (TT) and disulfide levels of the participants were measured with the fully automated spectrophotometric method described by Erel and Neselioglu [21]. The assay measured native thiol groups using a modified Ellman reagent, while dynamic disulfide bonds were reduced to free thiol groups by sodium borohydride (NaBH4), followed by formaldehyde to remove unused NaBH4. The total thiol amount was measured, and the disulfide bond amount was determined by taking half of the difference between the total thiol and native thiol amounts. The assay used chemicals such as reduced and oxidized glutathione, albumin, 2-mercaptoethanol, DTNB, hydrogen peroxide, chloramine-T, EDTA, NaBH4, TRIS, NaOH, methanol and formaldehyde. It employed an automated analyzer, completing the process within approximately 10 min without separation steps. Measurements were taken at 415 nm and 700 nm wavelengths. The disulfide/native thiol ratio (DNTR), disulfide/total thiol ratio (DTTR) and native thiol/total thiol ratio (NTTR) were calculated.

### 2.6. Statistical Analysis

The SPSS Statistics 22.0 program was utilized to calculate the means and standard deviations (SD) of the variables. To test the data normality, the Shapiro–Wilk test was used. Two-group comparisons were performed by using the independent sample *t*-test or the Mann–Whitney U test; more-than-two-group comparisons were performed by using the one-way ANOVA or Kruskal–Wallis tests, according to the data normality. We used post hoc tests and corrected the *p* values with Bonferroni correction. To perform correlation analysis, the Pearson test was applied for normally distributed values, and the Spearman test was applied for not-normally distributed values. The chi-square test was used to compare gender differences between groups. A *p* value lower than 0.05 was accepted as statistically significant for the two-group comparisons, lower than 0.0167 was accepted as statistically significant for the three-group comparisons, and lower than 0.0083 was accepted as statistically significant for the four-group comparisons.

## 3. Results

This study included 90 rosacea patients (57 females, 33 males) and 30 healthy controls (21 females, 9 males). The mean age of the patients was 52.25 ± 6.67 years, and the mean age of the controls was 51.80 ± 6.89 years. There was no statistically significant gender (*p* = 0.51) and age (*p* = 0.75) difference between the patients and controls.

Table 1 shows the comparison of NT, TT, disulfide, DNTR, DTTR and NTTR values between the patients and controls. The mean disulfide, DNTR and DTTR values were found to be significantly higher, and the mean NT, TT and NTTR values were found to be significantly lower in the rosacea patients compared to the controls (*p* < 0.001). TBUT and Schirmer results were significantly lower, and meiboscore and OSDI results were significantly higher in the rosacea patients compared to the controls (*p* < 0.01) (Table 1).

**Figure 4:**
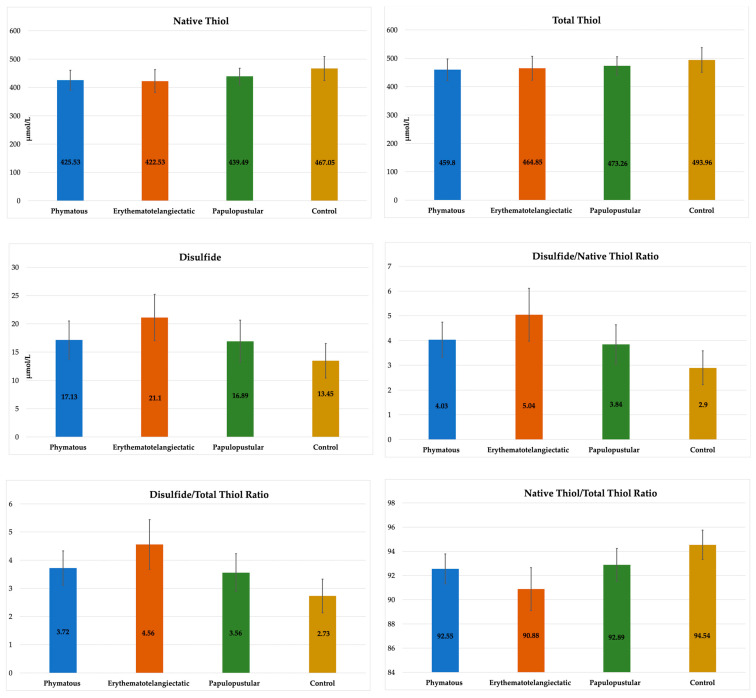
Comparison of biochemical values between rosacea subtypes and controls.

The rosacea patients were also examined by dividing them into subgroups according to their skin subtype (20 patients in the phymatous subtype, 40 patients in the erythematotelangiectatic subtype and 30 patients in the papulopustular subtype). There was no significant difference between the subgroups and controls considering age and gender (*p* > 0.05) (Table 2). Disulfide, DNTR, DTTR and NTTR values were significantly different in all three rosacea subtypes compared to the controls (*p* < 0.005) (Table 2, Figure 4). The NT value was significantly lower in the phymatous and erythematotelangiectatic subtypes compared to the controls (*p* < 0.005). The TT value was not different between any subtype and the controls (*p* > 0.0083). In the erythematotelangiectatic subtype, the mean disulfide, DNTR and DTTR values were significantly higher, and the mean NTTR value was significantly lower compared to the other subtypes (*p* < 0.002), (Table 2, Figure 4). No statistically significant difference was detected between the phymatous and papulopustular subtypes in terms of thiol/disulfide homeostasis (*p* > 0.05).

**Figure 5:**
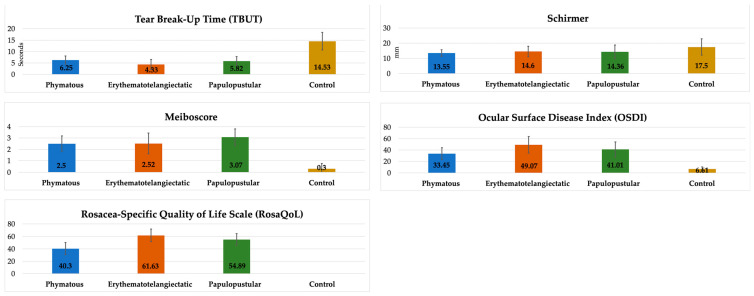
Comparison of clinical parameters between rosacea subtypes and controls.

When we evaluated rosacea-related clinical parameters according to skin subtypes, we found significantly lower TBUT and significantly higher RosaQol results in the erythematotelangiectatic subtype compared to the other subtypes (*p* < 0.0083). Interestingly, the RosaQol score in the phymatous subtype was significantly lower than that in the other subtypes, meaning that the phymatous patients complained less about their clinical findings. On the other hand, the meiboscore was higher in the papulopustular subtype compared to the other subtypes, but it did not reach a statistically significant level (*p* > 0.0083). All of the clinical parameters except Schirmer results were significantly different in all subtypes compared to the controls (*p* < 0.0083) (Table 2, Figure 5).

**Figure 6:**
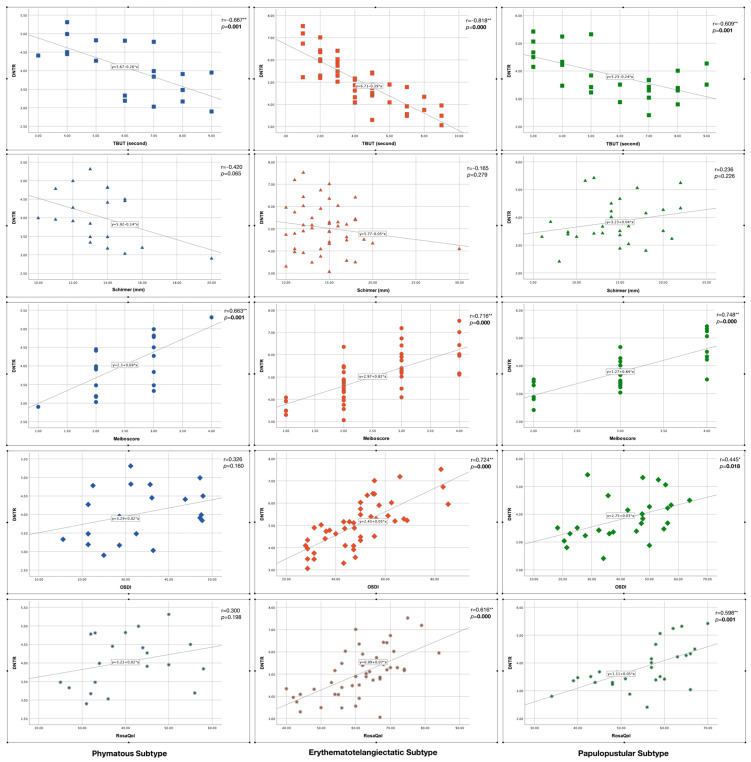
Correlation of disulfide/native thiol ratio (DNTR) with clinical parameters in rosacea subtypes. Statistically significant *p* values were indicated in bold font. * indicates statistically significance, ** indicates strong statistically significance.

Figure 6 shows the correlation analysis results between DNTR and clinical parameters in each skin subtype. There was a strong significant negative correlation between “DNTR and TBUT”, and there was a strong significant positive correlation between “DNTR and meiboscore” in all subtypes (*p* < 0.005). On the other hand, there were significant positive correlations between “DNTR and OSDI” and between “DNTR and RosaQol” in the erythematotelangiectatic and papulopustular subtypes (*p* < 0.05) but not in the phymatous subtype (*p* > 0.05). There was no significant correlation between DNTR and Schirmer in any subtype (*p* > 0.05).

## 4. Discussion

Rosacea is a chronic, inflammatory disease with an etiology still not fully elucidated [11]. According to recent studies, rosacea mostly affects people with compromised innate immune systems. The innate immune system is responsible for non-specific reactions to pathogens. Tissue infiltration by pathogens both causes and results in the activation of Toll-like receptors (TLRs), and these membrane-spanning proteins coordinate the innate immune response [29]. Particularly higher TLR4 levels have been demonstrated in rosacea skin and ocular specimens [16,30]. TLR2 and TLR4 activation triggers the release of mediators that initiate the inflammatory cascades, including cytokines, proteases, metalloproteinases and ROS [31].

ROS exert detrimental effects on fibroblasts, keratinocytes and endothelial cells. Thiols modify intracellular redox and protect keratinocytes from ROS. They are the first antioxidants to be ingested in an oxidative environment [32]. Endogenous and exogenous antioxidants are nucleophilic molecules that interact with ROS and eliminate their toxic effects on cell components. Changing the balance between antioxidants and oxidants can cause disruptions to cell homeostasis, initiate the emergence of pathogenic processes, and trigger chronic inflammation [33,34]. To investigate the association of this balance with various diseases, thiol-disulfide homeostasis has begun to be widely measured in recent years, particularly with the newly developed automated spectrophotometric method [18]. Some studies have shown that NT and TT levels are significantly low, especially in chronic inflammatory skin diseases including rosacea [21,22,32].

As the first in the literature, Şener et al. included 50 rosacea patients in a study and found that the average disulfide level and average DNTR and DTTR were significantly higher, and the average NTTR was significantly lower in rosacea patients compared to the control group [21]. Afterwards, Pektas et al. included 42 rosacea patients and showed similar results: higher DNTR and DTTR and lower NTTR in the patient group compared to the controls [22]. Additionally, they mentioned that different rosacea subtypes had no effect on oxidative stress markers. In our study, we included 90 rosacea patients with both skin and ocular involvement, and we found higher disulfide, DNTR and DTTR and lower NT, TT and NTTR in the patient group compared to the controls. However, when we investigated the rosacea patients according to their skin phenotype, the average disulfide, DNTR and DTTR were significantly higher, and the NTTR was significantly lower in the erythematotelangiectatic subtype compared to the other subtypes (papulopustular and phymatous). Our study results may be meaningful since they include more patients in the subgroups than the study by Pektas et al. Since oxidative stress particularly has been shown to cause vascular changes and inflammation in rosacea, it may play a more important role in the etiology of the erythematotelangiectatic subtype, where vascular changes and inflammation are more prominent.

On the other hand, Durmaz et al. included 40 rosacea patients and found that oxidative stress markers (serum total oxidant status (TOS), total antioxidant status (TAS), native thiol, total thiol and disulfide levels) did not differ statistically between the patients and controls [23]. However, they did not mention the patient’s skin phenotype. In most of the other studies investigating TAS and TOS in rosacea patients, it has been presented that serum TOS and oxidative stress index (OSI) levels are significantly higher, and TAS level is significantly lower in the patients [16,35]. Differently, Erdogan et al. found higher levels of TOS and TAS in rosacea patients (with no statistically significant differences in OSI) compared to the controls [36]. However, they mainly included patients with papulopustular skin phenotypes. Maybe all these contradictory results are specifically related to different skin subtypes.

Additionally, in our study, we investigated the ocular surface parameters that have been previously shown [16] to be closely related to oxidative stress and inflammatory factors in ocular rosacea patients and RosaQol that evaluates the quality of life of rosacea patients. We found significantly lower TBUT and Schirmer and significantly higher meiboscore, OSDI and RosaQol results in the patients compared to the controls. When we investigated the results according to skin subtypes, we found significantly lower TBUT and significantly higher RosaQol in the erythematotelangiectatic subtype compared to the papulopustular and phymatous subtypes, which might also be associated with the thiol-disulfide homeostasis results found in this current study. Interestingly, the RosaQol score in the phymatous subtypes were significantly lower than in the other subtypes, meaning that phymatous patients complained less about their clinical findings. Perhaps, in this way, they may have progressed to the phymatous level by being careless about treatment methods. On the other hand, the meiboscore was higher in the papulopustular subtype compared to the other subtypes, which may explain chalazion lesions (in ocular rosacea) that resemble papulopustular lesions of the skin rosacea. To better understand the oxidative stress effect in the subtypes, we investigated the correlation of DNTR with clinical parameters, and we found stronger correlations between DNTR and clinical parameters (particularly TBUT, OSDI and RosaQol) in the erythematotelangiectatic subtype compared to the other subtypes.

An effective treatment of rosacea is not yet available due to its complex etiopathogenesis [37]. Many treatment modalities are being tried. Recent studies indicate that some vitamins and minerals with antioxidant effects may offer safe and cost-effective alternatives for managing the symptoms of rosacea [38,39]. However, there is no clinical study measuring the impact of these supplements or other treatment interventions on the oxidative stress levels of rosacea patients.

On the other hand, oxidative stress has been shown to be associated with animal protein, fat and high carbohydrate diets [40,41]. A preclinical study investigating the effects of a high-fat diet on thiol disulfide homeostasis in rats revealed a significant redox imbalance in thiol disulfide metabolism at day 180 [42]. Environmental exposure to chemicals also causes oxidative stress and modulates the antioxidant system function in the body [43]. Therefore, factors such as diet, lifestyle or environmental exposure may have a potential impact on thiol/disulfide homeostasis in rosacea patients; however, there is no study that has addressed this topic yet.

Our study limitation may be not exploring the association of thiol-disulfide homeostasis with inflammatory factors, particularly in rosacea skin subtypes. Another limitation of our study may be the inclusion of patients from a certain geographical region and demographic group with similar environmental factors and lifestyle. Controlling this study across a wide geographic and demographic scope and environmental factors would have been challenging for us since rosacea is a disease influenced by many external and genetic factors. It would be interesting to investigate these differences in long-term and multicenter studies involving more patients and even observing the response to different treatments. Nevertheless, our study is the first in the literature to evaluate thiol-disulfide homeostasis and its association with clinical parameters among rosacea skin subtypes.

In conclusion, our study demonstrates that the thiol/disulfide balance shifted towards disulfides as an indicator of oxidative stress in the rosacea patients, and it was more prominent in the erythematotelangiectatic subtype. Rosacea-related clinical parameters that are associated with oxidative stress were also more prominent in the erythematotelangiectatic subtype. Therefore, with our study findings, it can be hypothesized that oxidative stress may play a more significant role in the pathophysiology of the erythematotelangiectatic subtype than the other skin subtypes. In order to better understand these outcomes, inflammatory and proinflammatory markers or their activators, such as TLR4, can be investigated in rosacea subtypes in future studies.

## Figures and Tables

**Table 1:** Comparison of demographic characteristics, thiol/disulfide homeostasis and clinical parameters between rosacea patients and healthy controls.

	Rosacea Patients (Mean ± SD)	Healthy Controls (Mean ± SD)	*p*
Age (Years)	52.25 ± 6.67	51.80 ± 6.89	0.75
Gender (male/female)	33/57	9/21	0.51
NT (µmol/L)	428.34 ± 36.46	467.05 ± 42.40	**<0.001**
TT (µmol/L)	466.30 ± 38.19	493.96± 43.31	**0.001**
Disulfide (µmol/L)	18.98 ± 4.34	13.45 ± 3.04	**<0.001**
DNTR	4.46 ± 1.07	2.89 ± 0.67	**<0.001**
DTTR	4.08 ± 0.90	2.73 ± 0.60	**<0.001**
NTTR	91.85 ± 1.79	94.44 ± 1.21	**<0.001**
TBUT (second)	5.18 ± 2.24	14.53 ± 3.84	**<0.001**
Schirmer (mm)	14.30 ± 3.56	17.50 ± 3.38	**0.004**
Meiboscore	2.68 ± 0.84	0.30 ± 0.46	**<0.001**
OSDI	43.03 ± 15.52	6.61 ± 3.84	**<0.001**
RosaQol	55.45 ± 13.33		

NT: native thiol, TT: total thiol, DNTR: disulfide/native thiol ratio, DTTR: disulfide/total thiol ratio, NTTR: native thiol/total thiol ratio, TBUT: tear breakup time, OSDI: ocular surface disease index, RosaQol: rosacea specific quality of life scale. A *p* value lower than 0.05 was accepted as statistically significant and indicated in bold font.

**Table 2:** Comparison of demographic characteristics, thiol/disulfide homeostasis and clinical parameters between rosacea skin subtypes and healthy controls.

	Phymatous Subtype				Erythematotelangiectatic Subtype			Papulopustular Subtype		Healthy Controls
	Mean ± SD	*p*	*p* *	*p* **	Mean ± SD	*p* ^†^	*p* ^‡^	Mean ± SD	*p* ^&^	Mean ± SD
Age (Years)	53.87 ± 5.82	0.27	0.45	0.33	51.80 ± 6.26	0.85	0.99	52.11 ± 7.76	0.87	51.80 ± 6.89
Gender (male/female)	8/12	0.71	0.81	0.47	14/26	0.89	0.66	11/19	0.58	9/21
NT (µmol/L)	425.53 ± 35.06	0.78	0.14	**0.001**	422.53 ± 40.39	0.58	**<0.001**	439.49 ± 28.59	0.04	467.05 ± 42.40
TT (µmol/L)	459.80 ± 38.01	0.65	0.19	0.02	464.85 ± 41.71	0.36	0.014	473.26 ± 32.13	0.29	493.96 ± 43.31
Disulfide (µmol/L)	17.13 ± 3.35	**0.001**	0.82	**0.004**	21.10 ± 4.10	**<0.001**	**<0.001**	16.89 ± 3.75	**0.003**	13.45 ± 3.04
DNTR	4.03 ± 0.71	**<0.001**	0.39	**<0.001**	5.04 ± 1.07	**<0.001**	**<0.001**	3.84 ± 0.79	**<0.001**	2.90 ± 0.68
DTTR	3.72 ± 0.61	**<0.001**	0.38	**<0.001**	4.56 ± 0.88	**<0.001**	**<0.001**	3.56 ± 0.67	**<0.001**	2.73 ± 0.60
NTTR	92.55 ± 1.23	**<0.001**	0.38	**<0.001**	90.88 ± 1.77	**<0.001**	**<0.001**	92.89 ± 1.35	**<0.001**	94.54 ± 1.21
TBUT (second)	6.25 ± 1.83	**0.006**	0.45	**<0.001**	4.33 ± 2.24	**0.007**	**<0.001**	5.82 ± 2.00	**<0.001**	14.53 ± 3.84
Schirmer (mm)	13.55 ± 2.16	0.21	0.46	**0.007**	14.60 ± 3.47	0.79	0.01	14.36 ± 4.44	0.02	17.50 ± 5.39
Meiboscore	2.50 ± 0.68	0.92	0.06	**<0.001**	2.52 ± 0.91	0.02	**<0.001**	3.07 ± 0.72	**<0.001**	0.30 ± 0.47
OSDI	33.45 ± 10.56	**<0.001**	0.09	**<0.001**	49.07 ± 14.54	0.03	**<0.001**	41.01 ± 13.17	**<0.001**	6.61 ± 3.84
RosaQol	40.30 ± 9.84	**<0.001**	**<0.001**		61.63 ± 10.06	**0.016**		54.89 ± 9.50		-

NT: native thiol, TT: total thiol, DNTR: disulfide/native thiol ratio, DTTR: disulfide/total thiol ratio, NTTR: native thiol/total thiol ratio, TBUT: tear breakup time, OSDI: ocular surface disease index, RosaQol: rosacea specific quality of life scale. *p*: Comparisons between phymatous and erythematotelangiectatic groups. *p* *: Comparisons between phymatous and papulopustular groups. *p* **: Comparisons between phymatous and control groups. *p* ^†^: Comparisons between erythematotelangiectatic and papulopustular groups. *p* ^‡^: Comparisons between erythematotelangiectatic and control groups. *p* ^&^: Comparisons between papulopustular and control groups. A *p* value lower than 0.0083 was accepted as statistically significant after Bonferroni post hoc test correction for four-group comparisons. A *p* value lower than 0.0167 was accepted as statistically significant after Bonferroni post hoc test correction for three-group comparisons. Statistically significant *p* values were indicated in bold font.

## Data Availability

The data supporting the findings of this study are available from the corresponding author upon reasonable request.
